# Emerging roles of inflammation-mediated endothelial–mesenchymal transition in health and disease

**DOI:** 10.1186/s41232-021-00186-3

**Published:** 2022-02-07

**Authors:** Yasuhiro Yoshimatsu, Tetsuro Watabe

**Affiliations:** 1 Division of Pharmacology, Graduate School of Medical and Dental Sciences, Niigata University, Niigata, Japan; 2 Department of Biochemistry, Graduate School of Medical and Dental Sciences, Tokyo Medical and Dental University (TMDU), Tokyo, Japan

**Keywords:** Endothelial–mesenchymal transition (EndoMT), Epithelial–mesenchymal transition (EMT), Transforming growth factor-β (TGF-β), Inflammation, Fibroblast, Myofibroblast, Cancer-associated fibroblast (CAF)

## Abstract

Endothelial–mesenchymal transition (EndoMT), a cellular differentiation process in which endothelial cells (ECs) lose their properties and differentiate into mesenchymal cells, has been observed not only during development but also in various pathological states in adults, including cancer progression and organ/tissue fibrosis. Transforming growth factor-β (TGF-β), an inflammation-related cytokine, has been shown to play central roles in the induction of EndoMT. TGF-β induces EndoMT by regulating the expression of various transcription factors, signaling molecules, and cellular components that confer ECs with mesenchymal characteristics. However, TGF-β by itself is not necessarily sufficient to induce EndoMT to promote the progression of EndoMT-related diseases to a refractory extent. In addition to TGF-β, additional activation by other inflammatory factors is often required to stabilize the progression of EndoMT. Since recent lines of evidence indicate that inflammatory signaling molecules act as enhancers of EndoMT, we summarize the roles of inflammatory factors in the induction of EndoMT and related diseases. We hope that this review will help to develop therapeutic strategies for EndoMT-related diseases by targeting inflammation-mediated EndoMT.

## Background

The vascular system is essential for embryonic development and the homeostasis of tissues and organs during adult life. Aberrant vascularization is associated with a number of diseases, including cancer, atherosclerosis, hypertension, and retinopathy. Endothelial cell (EC) activation and homeostasis are regulated by the coordinated activity of cellular signaling pathways and networks of transcription factors (TFs). In vivo analyses using genetically modified mice have shown that the deletion of TGF-β signaling components in ECs leads to the abnormal formation of the primitive vascular plexus and decreased vessel wall integrity caused by abnormal capillary vessel formation or impaired differentiation, as well as the recruitment of vascular smooth muscle cells (SMCs), during embryonic development. These results could explain the significance of these components in the pathogenesis of certain hereditary human vascular diseases. It is clearly one of the most critical factors as an endothelial–mesenchymal transition (EndoMT) inducer. Here, we briefly summarize TGF-β signaling and EndoMT.

### TGF-β superfamily

**Fig. 1:**
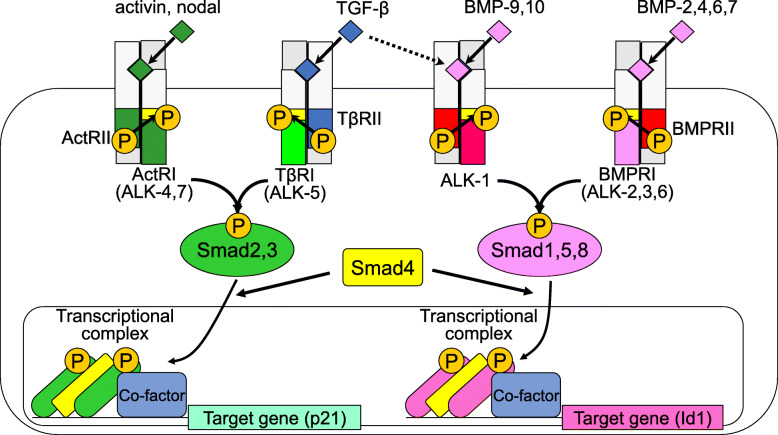
TGF-β family signaling pathways

TGF-β is produced as a latent complex with no activity, requiring an activation step to exert its function as an active molecule [1]. In mammals, there are three isoforms of TGF-β (TGF-β1–3), and approximately 30 other structurally similar proteins that constitute the TGF-β superfamily, including activin, nodal, and bone morphogenetic protein (BMP), many of which are strongly involved in cell differentiation, cell proliferation, and cell death in vitro (Fig. 1). The typical biological effects of TGF-β on blood vessels include the inhibition of proliferation, the induction of apoptosis, the promotion of matrix production and degradation, and the resolution of inflammatory responses during vascular injury.

### Receptors for TGF-β

The TGF-β family transduces signals by binding to two types of receptors, TGF-β type I (TβRI, also known as activin receptor-like kinase-5 (ALK-5)) and type II (TβRII) receptors (Fig. 1). TβRII specifically binds to the three isoforms of TGF-β in the extracellular domain, and two pairs of TβRII and TβRI bind to TGF-β to form a heterotetramer. In addition to TβRI, there are six other type I receptors, which act as receptors for activin, nodal, and BMPs. Activin and nodal bind to the activin type II receptor (ActRII), while BMPs bind to the BMP type II receptor (BMPRII).

### Smad intracellular signaling

Intracellular signaling factors of the TGF-β family are mainly mediated by a group of factors called Smads. Eight types of Smads have been identified in mammals and are classified as receptor-regulated Smad (R-Smad), common-mediator Smad (Co-Smad), and Inhibitory Smad (I-Smad, not shown in Fig. 1). The R-Smads acting downstream of TGF-β are mainly Smad2 and Smad3, while the R-Smads acting downstream of BMPs are mainly Smad1, 5, and 8. The Co-Smads found in mammals are only Smad4, while the I-Smads are Smad6 and Smad7. When TβRI is activated by TβRII, R-Smads are phosphorylated. As a result, R-Smad forms a complex with Co-Smad and translocates into the nucleus, where it binds to various TFs and cofactors to regulate the transcription of target genes. I-Smad expression increases upon stimulation by TGF-β and/or BMPs and then suppresses TGF-β and/or BMP signaling by negative feedback.

**Fig. 2:**
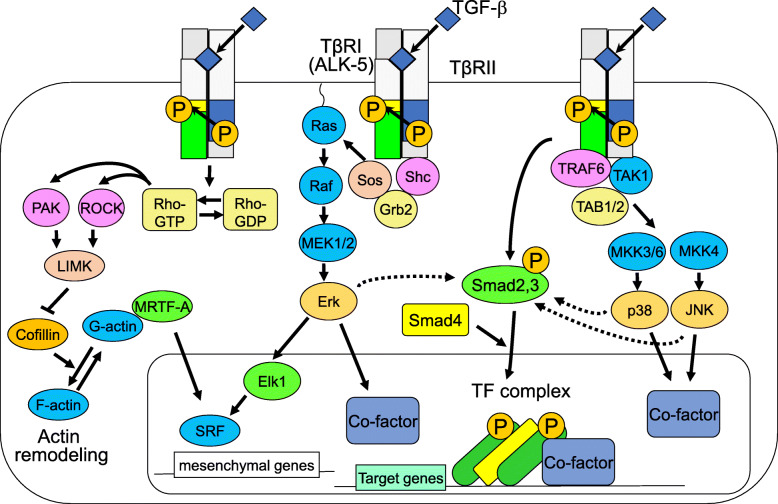
TGF-β-mediated non-Smad pathways

The TGF-β receptor activates not only the Smad signaling pathway, but also non-Smad signaling pathways (Fig. 2). These pathways include mitogen-activated protein kinase (MAPK) pathways, such as the Erk, JNK, and p38 MAPK, and PI3K-Akt pathways (not shown in Fig. 2), and Rho-like GTPases. These pathways are non-canonical pathways, called non-Smad pathways, which often cooperate with Smad signaling to regulate cell proliferation, motility, and differentiation. For example, the activation of Erk, p38, and JNK can even lead to the regulation of Smad activity.

### EMT and EndoMT

The epithelial–mesenchymal transition (EMT) is a phenomenon in which epithelial cells differentiate into mesenchymal cells and is widely recognized as a mechanism of malignant transformation of cancer, in which epithelial-derived cancer cells acquire migratory properties and invade blood vessels. In cardiac development, a molecular mechanism similar to EMT has been shown to occur in ECs. In recent years, it has been clarified that a similar phenomenon occurs in vascular ECs with various pathological conditions, which has been defined as EndoMT and is now widely recognized. In particular, TGF-β, which is abundantly produced in chronic inflammation and the tumor microenvironment, is now known to be a potent inducer of not only EMT but also EndoMT, and much more attention has been focused on how it acts on ECs and how EndoMT progresses.

**Fig. 3:**
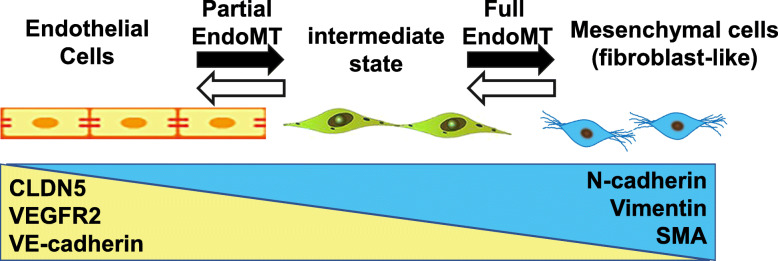
Endothelial-mesenchymal transition (EndoMT)

When ECs are exposed to EndoMT inducers, such as TGF-β, intercellular adhesion is weakened due to the decreased expression of endothelial-specific cell adhesion markers, such as vascular endothelial (VE)-cadherin (Fig. 3). On the other hand, when the expression of mesenchymal cell markers is increased, the cells acquire mesenchymal properties, such as the enhancement of the migratory ability of the cells. In in vivo analyses, Zeisberg et al. observed EndoMT in a mouse tumor xenograft model using cell lineage tracing, a method used to track the origin of the cells during cell differentiation in order to identify cells that have undergone EndoMT [2]. They used transgenic mice expressing *Tie2*-promoter-driven Cre recombinase crossed with *R26Rosa-lox-Stop-lox-LacZ* mice to label ECs and hematopoietic cells, which would later become fibroblasts (cancer-associated fibroblasts (CAFs) in this case). It has been shown that up to 40% of CAFs, which are thought to be malignant in cancer [2], are derived from ECs. When transgenic mice expressing *Tie1*-promoter-driven Cre recombinase were used, up to 30% of fibroblasts in the process of cardiac fibrosis were derived from ECs [3]. ECs have been reported to undergo EndoMT not only in cancer and organ/tissue fibrosis but also in other pathological conditions, such as atherosclerosis, pulmonary hypertension (PH), and diabetes. In addition to its role as a CAF during cancer progression, it has been suggested to promote angiogenesis and metastasis. In the process of fibrosis, in addition to differentiation into activated myofibroblasts and production of fibrosis-promoting factors, EndoMT is thought to play a role in tissue stiffening by expressing extracellular membrane (ECM) proteins. In atherosclerosis, EndoMT plays a role in narrowing blood vessels by neointimal fibrosis and promoting vascular calcification. In PH, EndoMT induces structural and functional pulmonary vascular changes, and in diabetes mellitus, vascular fibrosis and damaged blood vessels have been observed.

ECs in the resting state usually do not interact with leukocytes. This is due to the lack of leukocyte-interactive proteins on the endothelial surface, such as P-selectin, E-selectin, ICAM1, and VCAM1. During acute inflammation, inflammatory cytokines induce the expression of these proteins, with the exception of a rapid transport system for P-selectin from intracellular storage granules to the plasma membrane. In inflamed ECs, intercellular junctions become disrupted, leading to elevated vascular permeability until the inflammation is resolved. In chronic inflammation, various factors, such as inflammatory cytokines, reactive oxygen species, and oxidized low-density lipoprotein (LDL), persistently activate ECs, leading to aberrant vascular relaxation, increased leukocyte adhesion, increased endothelial permeability, and even the generation of a pro-thrombotic state. These types of assaulting factors induce endothelial dysfunction, accompanied by the observation of EndoMT.

Before introducing studies on inflammation-mediated EndoMT, we summarize some fundamental information on EndoMT in the following sections.

### EndoMT markers: EC and mesenchymal cell markers

Whether cells that have completely differentiated into mesenchymal cells are derived from ECs cannot be discriminated against. Therefore, in a stricter sense, only mesenchymal cells that continue to express EC markers have been analyzed in many studies. In this respect, as described above, Zeisberg et al. and other groups performed cell lineage tracing studies to analyze EndoMT [2]. Although several representative EC markers are widely accepted, those that have been commonly used for EndoMT analysis include VE-cadherin, PECAM1, vWF, VEGFR2, and TIE2 (Table 1). In particular, TIE2 has been used as an EC marker in many studies, but is also expressed in hematopoietic lineage cells. EC markers whose expression is restricted to ECs, such as VE-cadherin, have been preferentially used in recent years. On the other hand, mesenchymal cell markers include smooth muscle α-actin (SMA/ACTA2), smooth muscle 22α (SM22α/TAGLN), N-cadherin/CDH2, fibronectin (FN1), type I collagen, and type III collagen (Table 1). As for mesenchymal cell markers, many factors are common in EMT. The expression of Snail/SNAI1, Slug/SNAI2, and Twist1 in ECs has been reported to enhance EndoMT [4–6], and these factors may play a central role in promoting EndoMT. High levels of ZEB1 expression in the tumor ECs (TECs) of lung cancer have been shown to exacerbate the tumor microenvironment, including increased metastatic potential and decreased anti-tumor immunity driven by EC-secreted TGF-β [7]. However, the observation of EndoMT in this study was not mentioned. Twist2 and ZEB2 have been reported to be upregulated during EndoMT; however, their direct involvement has not been shown. EndoMT-related markers are increasingly being used as evidence for EndoMT, and we will describe several of these within this review.

**Table 1:** List of EndoMT-related markers and inflammation-related genes in articles introduced in this review

Inducer/trigger	Inflammation signal	EC gene	Mesenchymal gene		Transcription factor
BMP9	CCL2	APLN	ACTA2/SMA	MMP14	HES1
MMP14/MT1-MMP (macrophage)	CCL3	CDH5/VE-cadherin	CDH2	MMP2	HEY1
TGFB1	CXCL2	ERG	CNN1/SM-calponin	MMP9	HEY2
TGFB2 (endothelial)	ICAM1	FGFR1	COL12A1	NOTCH3	MKL1/MRTFA
	IFNG	FLT4 (lymphatic EC)	COL1A1	PCOLCE	SNAI1
	IL1B	FSTL3	COL1A2	PDGFRB	SNAI2
	IL4	KDR	COL2A1		TWIST1
	IL6	lncRNAH19	COL3A1	S100A4/FSP1	TWIST2
	JAK	LYVE1 (lymphatic EC)	COL6A1	SERPINE1/PAI1	ZEB1
	RELA	MIR126	CTGF	TAGLN	ZEB2
	STAT3	MIRLET7/let-7	FAP	TGFBI	
	TNFA	NOS3/eNOS	FN1	TNC	
	VCAM1	PDPN (lymphatic EC)	LAMC2	VIM	
		PECAM1/CD31	LY6A/Sca1		
	(EndICLT marker)	PROX1 (lymphatic EC)			
	C1QA	PTGDS/L-PGDS			
	C1QB	SCL (EC/hematopoietic)	ATG5^low^ (endothelial)		
	C5AR1	TIE1	ATG16L^low^ (endothelial)		
		TIE2 (EC/hemopoietic)	TRP53^low^ (endothelial)		
		VWF			
		WNT5A			

Further insights into EndoMT are given in Tables 1, 2, and 3, below.

**Table 2:** Mouse/rat models or human samples used in the studies introduced in this review

Model/sample type	References
Cardiac fibrosis	
Aortic banding/ascending aortic constriction	[3, 8]
HFD-induced cardiac fibrosis	[9]
Myocardial infarction (ligation of the left anterior descending artery)	[8, 10–12]
Myocardial infarction (distal left coronary artery ligation)	[13]
Lung fibrosis	
Tumor-driven fibrosis (*Kras* and *Trp53* deficiency)	[14, 15]
Bleomycin- and hypoxia-induced PH	[16, 17]
Monocrotaline (MCT)-induced pulmonary hypertension	[16, 17]
MCT-induced PAH	[18]
Radiation-induced fibrosis	[14, 15, 19]
Sugen or combination of sugen and hypoxia-induced PAH	[20]
Human lung cancer sampes (small-cell, large-cell, and squamous cell carcinoma and adenocarcinoma)	[14]
PAH patient-derived MVEC	[21]
Liver fibrosis	
Carbon Tetrachloride CCL4 liver injury model	[22]
EC-specific deletion of *Erg*	[22]
Patient-derived livers with end-stage liver diseases including non-alcoholic steatohepatitis (NASH), primary biliary cholangitis (PBC), and alcohol-related liver disease (ALD)	[22]
Renal fibrosis	
HFD-induced renal fibrosis in EC-specific deletion of *Atg5*	[9]
Orthotopic left kidney transplantation model	[23]
Serum samples from patients with chronic allograft dysfunction	[23]
Atherosclerosis	
*ApoE3* Leiden mice (hyperlipoproteinemia/hypercholesterolemic model)	[24]
High-fat diet-induced atherosclerosis model in *ApoE* KO mice	[25–28]
Disturbed flow-induced atherosclerosis after partial carotid ligation surgery	[29]
Human samples of atherosclerotic coronary arteries	[30]
Human advanced atherosclerotic samples	[24]
Transplant arteriopathy, femoral artery injury model, vein graft model	[31]
Diabetes (streptozotocin-induced hyperglycemia)	[32]
Tail lymphedema model	[33]
Tumor EC-derived CAF	
Syngeneic graft tumor	[2, 34]
Spontaneous mammary tumors	[35]
Syngeneic graft tumor in EC-specific deletion of *Ramp2*	[36]
Syngeneic graft tumor in EC-specific deletion of *Ptgds*	[37]
Radiation-induced EndoMT in *Kras* and *Trp53*-driven spontaneous lung tumor	[15]
Samples from patients with lung cancer who received radiotherapy	[15]

**Table 3:** In vitro models in the studies introduced in this review. EC subtypes or other cell types co-cultured with EC/cultured in supernatant from EC

Culture model and EC subtype	References
Human	
Aortic EC (HAoEC/HAEC)	[24, 29, 32, 38, 39]
Coronary EC	[3]
Coronary artery EC (HCAEC)	[13, 39, 40]
Dermal lymphatic EC (HDLEC)	[33, 41]
Endothelial colony-forming cells (ECFC)	[21]
Hepatic sinusoidal EC (HSEC)	[22]
Juvenile foreskin lymphatic EC	[42]
Microvascular EC (HMVEC)	[5, 9]
Umbilical vein EC (HUVEC)	[8, 17, 26, 32, 36, 39, 40, 43–45]
Umbilical artery EC (HUAEC)	[34, 46]
Pulmonary aortic ECs/human pulmonary artery ECs (HPAECs)	[6, 14, 21, 39, 47]
Pulmonary microvascular endothelial cell (HPMEC)	[14]
Pulmonary microvascular endothelial cell (MVEC) from PAH patients	[21]
Renal glomerular endothelial cell (HRGEC)	[23]
Skin microvascular EC (HMEC)	[40]
Mouse	
Cardiac EC	[27]
Lung EC	[2, 28]
EC isolated from infarcted hearts	[10]
ES-derived EC (MESEC)	[4]
Mesenteric LEC immortalized by SV40 tsA58T antigen	[33]
TEC derived from spontaneous mammary tumors	[35]
TEC derived from human melanoma xenograft	[48]
TEC derived from syngeneic melanoma and lung carcinoma	[37]
Other animal source	
Rat pulmonary arterial EC (PAEC)	[18]
Bovine aortic EC (BAEC)	[28]
Co-culture/culture with supernatant	
Oral cancer cell culture in supernatant from HUAEC undergoing EndoMT	[49]
Co-culture of mouse aortic endothelial cell (MAEC) with LPS-activated BMDM (macrophage)	[12]
Co-culture of HUVEC with primary melanoma cells collected from tumors	[36]

### EndoMT and EndoMyoT

There is a strict distinction between SM22A/TAGLN and SMA/ACTA2, which are considered as markers for mesenchymal cells exhibiting EndoMT/EMT, with SMA, in particular, being recognized as a myofibroblast marker [48, 50, 51]. During the differentiation of vascular SMCs, the expression of SM22A precedes that of SMA, and SMA expression is upregulated at a later stage. In recent years, among various mesenchymal cell markers, only SMA has been considered as a myofibroblast marker, and differentiation into SMA-expressing fibroblasts (myofibroblasts) has been assessed as EndoMyoT (endothelial–myofibroblast transition).

### EndoMT in development

In embryonic heart development, EndoMT occurs when endocardial cells, which are specialized ECs that form the innermost layer of the heart wall, form endocardial cushion [52]. This is not observed in Tgfb2 knockout (KO) mice [53]. In addition, when the receptor for BMP signaling is defective in ECs, the formation of atrioventricular valves in the heart does not take place. Atrioventricular valve formation is regulated by SNAI1 and Notch signaling [54].

Vascular development and regeneration are accompanied by vessel elongation, which is promoted by EC migration. During this process, the expression of EMT-related TFs, such as Slug, is upregulated. Thus, EndoMT is thought to exhibit reversible changes during vascular remodeling, such as transient occurrence and return to ECs [43]. Recently, the transit state of EndoMT, which is referred to as endothelial mesenchymal activation (EndMA), was evaluated as an important factor for myocardial infarction (MI) [8]; for more information, see the “Roles of transient EndoMT in EC plasticity” section.

## Inflammation-mediated EndoMT

### Roles of EndoMT in organ fibrosis

The accumulation of activated fibroblasts and myofibroblasts in each organ during tissue remodeling causes structural changes, leading to tissue dysfunction. The prevention and treatment of fibrosis are crucial clinical issues. In the process of the fibrosis of the heart, kidney, lung, and liver, the accumulation of EC-derived mesenchymal cells through EndoMT has been reported to occur at a considerable rate, although it was previously thought that fibrosis was promoted mainly through EMT and activation of resident fibroblasts.

Angpt2 is an antagonist of Angpt1/Tie2 signaling, which plays an important role in stabilizing vascular structures [55]. Lee et al. found that Angpt2 is highly expressed in the ECs of the border zone of the infarct area in the heart after MI, with a concomitant marked increase in FOXO1 expression, which positively regulates Angpt2 expression in ECs [10]. Angpt2 induces pericyte detachment from ECs of the infarcted border zone of the ischemic heart by antagonizing Angpt1/Tie2 signaling. Angpt2 exacerbates vascular leakage, cardiac hypoxia, and infarction. Angpt2 also aggravates vascular inflammation and myocardial remodeling after MI. Conversely, the EC-specific deletion of *Angpt2* using inducible *Cdh5*-Cre mice reduced vascular leakage, improved microvascular perfusion and tissue oxygenation, and ameliorated the expression of adhesion molecules and neutrophil infiltration in the ischemic heart. In the chronic remodeling phase (2-3 weeks) after MI, both endothelial- and macrophage-derived Angpt2 continuously promote abnormal vascular remodeling and proinflammatory macrophage polarization, accelerating cardiac hypoxia and inflammation. The deletion of *Angpt2* suppresses the upregulated expression of EndoMT-related genes, such as *Col12a1*, *Fstl3*, and *Pcolce*, as indicated by the gene set enrichment analysis (GSEA) of isolated ECs from infarct hearts. The genetic or pharmacological inhibition of Angpt2 ameliorates post-ischemic infarct size and cardiovascular remodeling.

Nur77, an orphan nuclear receptor, has previously been shown to be upregulated in MI [11, 56]. In a mouse model of myocardial fibrosis after MI by distal left coronary artery ligation, *Nur77* deficiency in mice showed exacerbated cardiac function and cardiac fibrosis after MI, with a concomitant increase in the expression of *Col1a1* and FSP1, and a decreased expression of *Pecam1 and Nos3* [13]. Mechanistically, treatment with TGF-β2 in combination with interleukin-1β (IL-1β) in human coronary artery EC (HCAECs) was found to downregulate the expression of PECAM1 and upregulate the expression of TAGLN. Nur77 knockdown (KD) enhanced the phosphorylation of NF-κB, suggesting that Nur77 suppresses EndoMT by inhibiting the NF-κB-mediated inflammatory response in ECs.

Macrophages play important roles in removing necrotic cellular debris, and damaged ECM from the tissues and recruiting other immune cells through the secretion of pro-inflammatory cytokines, including tumor necrosis factor-α (TNF-α), IL-1β, and IL-6, which further sustain inflammation. In the MI model induced by ischemic injury using permanent ligation of the left anterior descending coronary artery, macrophages that produce such pro-inflammatory cytokines have been shown to contribute to cardiac dysfunction after MI through EndoMT [12]. The expression of matrix metalloproteinase (MMP) 14 was upregulated after MI. Additionally, the expression of *Mmp2* and *Col1a1*, both of which are substrates of MMP14, was also upregulated after MI. Macrophage-specific *Mmp14* deletion in *Lyz2*-Cre mice indicated better preservation of cardiac function and reduced wall-motion abnormalities in the left ventricle, most likely due to reduced hypoxia, suggesting smaller infarcts by this deletion. MMP14 has been shown to be a key regulator of latent TGF-β activation. TGF-β activity was detected in lipopolysaccharide (LPS)-activated bone marrow-derived macrophages (BMDMs) of WT mice but not in macrophage-specific *Mmp14* KO mice. The deletion also reduced TGF-β1-pSMAD2 signaling in cardiac ECs, myofibroblasts, and vascular SMCs after MI. Inducible *Cdh5*-Cre-mediated cell lineage tracing indicated enhanced EndoMT, suggested by the downregulation of *Pecam1*, *Cdh5*/VE-cadherin, *Kdr*, and *Tie2*, and the upregulation of *Cdh2*, *Tagln*, *Col1a1*, *Col1a2*, *Col1a3*, *Zeb2*, and *Snai1* in PECAM1+PDGFRB+ (platelet-derived growth factor receptor β+) cells as EndoMT cells in the infarcted myocardium. However, deletion in mice showed an approximately 50% reduction in the EndoMT ratio. Mechanistically, co-culture experiments of purified mouse aortic ECs (MAECs) with LPS-activated BMDMs from macrophage-specific *Mmp14* KO mice demonstrated that the ratio of PECAM1+ MAECs was increased and that of SMA+ MAECs decreased. Finally, enhanced EndoMT expression was reversed by treatment with TGF-β1-neutralizing antibodies. Taken together, impairment of MMP14 function in macrophages after MI reduces macrophage-mediated EndoMT and fibrosis, limits left ventricle remodeling, and preserves cardiac function.

In pulmonary fibrosis, HSPB1, also known as HSP27, which has a protective role against cellular stress [57, 58], has previously been reported as a regulator of fibrosis through EMT [59]. Choi et al. found that HSPB1 functions as a specific regulator of EndoMT and examined the effects of endothelial HSPB1 on the development of pulmonary fibrosis and lung tumor progression (see section 4 “Roles of EndoMT in Tumor EC”) [14]. The KD of HSPB1 in human pulmonary microvascular ECs (HPMEC) induced EndoMT, characterized by the downregulation of CDH5, VEGFR2, and PECAM1 and the upregulation of SMA, as well as TFs *ZEB1/2*, and *TWIST1/2*. In contrast, the forced expression of HSPB1 suppressed TGF-β-induced EndoMT in HPMECs. The group previously showed that EndoMT in human aortic ECs (HAECs) is associated with radiation-induced vascular fibrosis [19]. The irradiation of HPMECs increased SMA expression in HSPB1-knocked-down cells. In lung tissue samples from mice with radiation-induced pulmonary fibrosis, cytokines in the serum included significant levels of IL-1β, SDF-1, and PAI-1. In radiation-induced lung fibrosis, in which HSPB1 is overexpressed in mice, collagen deposition was induced. The ratios of EndoMT (SMA+ PECAM1+ EC; SMA and PECAM1-double-positive EC) were significantly increased.

Fibrotic tissue has also been observed in PH or pulmonary arterial hypertension (PAH). The CD44 variant (CD44v) isoform, CD44v8-10, is known to bind and to stabilize the cystine transporter subunit (xCT), producing reduced glutathione, thereby enhancing the antioxidant defense of cancer stem cells [60, 61]. CD44v was predominantly expressed on EndoMT-like cells in the neointimal layer of pulmonary arterioles affected by PAH, while the isoform was not detected in normal pulmonary vessels [20]. The downregulation of PECAM1 and the upregulation of EndoMT-related markers, such as *SNAI1/2*, *MMP2/9*, and SMA, were observed in human pulmonary artery ECs (HPAECs) treated in combination with TNF-α, IL-1β, and TGF-β. The treated HPAECs induced the expression of CD44v and xCT. Furthermore, CD44v formation was regulated by splicing factors, serine/arginine-rich splicing factor (SRSF)-1 and -3, instead of ESPR1, which is well known to induce a splicing switch from the CD44 standard form to CD44v, particularly to CD44v8–10 in cancer cells. CD44v+ EndoMT+ HPAECs was found to result in the downregulation of *APLN* and *NOS3* and the upregulation of anti-apoptotic factors (*BCL2, BCLXL*), as well as antioxidative stress (*GPX1*), cell cycle arrest inducers (p16/*CDKN2A*, p21/*CDKN1A*, Trp53/*TP53*) and proinflammatory cytokines (*TNFA*, *IL1B*, *TGFB*). These cells also have much more reactive oxygen species (ROS). In turn, the intracellular redox balance was shifted toward more reducing conditions in CD44v+xCT^hi^ cells, consistent with higher intracellular glutathione levels. Sulfasalazine, an xCT-mediated cystine transport inhibitor, significantly suppressed the growth of CD44v+xCT^hi^ EndoMT HPAEC and the production of IL-6 from the EndoMT cells, suggesting that the survival of the EndoMT cells is highly dependent on xCT function. Finally, in the mouse model of PAH, which was induced by the combinatorial treatment of hypoxia and Sugen (SU5416, a VEGF receptor blocker), sulfasalazine administration significantly reduced the muscularization of peripheral pulmonary arteries (vascular remodeling) and the proportion of CD44v+ vessels, along with a decrease in EndoMT cells and decreased expression levels of inflammation-related genes, such as *TNFA*, *IL1B*, and *IL6*.

DPP-4 is a serine protease that selectively cleaves N-terminal dipeptides from substrates such as glucagon-like peptide-1 (GLP-1), resulting in a short half-life. DPP-4 inhibition by sitagliptin, an approved agent for the treatment of patients with type 2 diabetes, has previously been shown to alleviate pulmonary vascular remodeling in monocrotaline (MCT)-induced PH rats [16]. GLP-1 expression was upregulated in the lungs of MCT-challenged rats [47]. Treatment with sitagliptin significantly inhibited the MCT-induced upregulation of GLP-1 in lung tissues, while this effect was completely blocked by the GLP-1 receptor antagonist, exendin-3. Next, the GLP-1 analog liraglutide, an agent approved for the treatment of patients with type 2 diabetes, alleviated MCT-induced PH and suppressed MCT-induced inflammation and EndoMT in pulmonary arteries. The inflammation in the model seemed to be mediated by the infiltration of CD68+ macrophages and mast cells, as suggested by the increased expression of TGF-β1, IL-1β, TNF-α, and IL-6. Liraglutide blocked the upregulation of these cytokines. In the MCT-affected lung tissues, the downregulation of VE-cadherin and upregulation of FN1 and vimentin were also observed, suggesting that MCT-induced PAH accompanies EndoMT. In the bleomycin- and/or hypoxia-induced PH model, liraglutide partially alleviated PH progression and reduced the bleomycin- and hypoxia-upregulated expression of GLP-1. Mechanistically, liraglutide suppressed EndoMT in human umbilical vein ECs (HUVECs) induced by treatment with TGF-β and IL-1β. The phosphorylation of Smad2 and Erk was diminished by liraglutide, suggesting that both Smad and non-Smad pathways were blocked by liraglutide (Figs. 1 and 2). This effect of liraglutide was partially inhibited by the GLP-1 receptor antagonist, exendin-3. Taken together, these findings indicate that GLP-1 could be a novel therapeutic method for the treatment of PH. Recently, a meta-analysis showed that DPP4 inhibitors may improve mortality in COVID-19 disease [62]. The pathogenesis of COVID-19 is known to involve not only viral infection of tracheal and alveolar epithelial cells but also pulmonary ECs. Metabolic syndromes including diabetes accompany endothelial dysfunction. Type II diabetic patients affected by COVID-19 have a high mortality rate and are prone to develop severe COVID-19 symptoms. The severe symptoms are often thought to be mediated by a so-called “cytokine storm,” an uncontrollable state of cytokine releases. Since EndoMT cells more likely produce inflammatory cytokines by themselves, suggesting a vicious cycle, EndoMT cells may be involved in the enhancement of cytokine storms. Therefore, until drugs effective against COVID-19 viruses are developed, drugs for type II diabetes such as DPP4 inhibitors and GLP-1 may protect COVID-19 patients from severe symptoms by improving endothelial damage and inhibiting EndoMT.

*miR-181b*, a member of the *miR-181* family, has previously been shown to have a beneficial effect on vascular ECs by inactivating NF-кB via the regulation of importins [17, 63]. In the MCT-induced PAH rat model, in which EndoMT was induced, the expression of *miR-181b* was downregulated [64]. The lentiviral delivery of *miR-181b* ameliorated PH, pulmonary arterial hypertrophy, and right ventricular remodeling. The forced expression of *miR-181b* in MCT-challenged lung tissues rescued the downregulation of VE-cadherin and upregulation of SMA, CDH2, VIM, and collagen I. Mechanistically, rat PAECs treated with TNF-α, TGF-β1, and IL-1β significantly decreased the expression of *miR-181b*. The treated cells showed increased expression of TβRI and Endocan, both of which are targets of *miR-181b*. Endocan has previously been reported to regulate cell adhesion and growth. The KD of *miR-181b* further enhanced the inflammatory cytokine-induced EndoMT in rat PAECs. These data suggest that *miR-181b* plays a pivotal role in maintaining pulmonary ECs to protect from PAH by suppressing EndoMT.

Smaller pulmonary arteries, which are lined with highly specialized microvascular ECs (MVECs), are the principal sites of vascular remodeling in PAH [18]. Mutations in BMPRII are known to cause hereditary PAH. BMP-9, one of the ligands of BMPRII, binds to a BMPRI, activin receptor-like kinase 1 (ALK-1), whose expression is selectively localized to ECs. Szulcek et al. found aberrant BMP-9 responses in MVECs between healthy controls and patients with PAH, characterized by the significant upregulation of BMPRII and its target genes ID1/ID3 in PAH cells [21]. RNA sequencing analysis of BMP-9-stimulated PAH MVECs revealed that BMP-9 is a potent inducer of EndoMT transcriptional signaling, presumably mediated by *SNAI1/2* and *HEY1/2*. The long-term treatment of PAH MVECs with BMP-9 induced EndoMT, suggested by elongated F-actin stress fiber formation and decreased EC barrier function, both of which are characteristic of mesenchymal cell types. Based on a GSEA suggesting upregulation in IL-6, JAK, and STAT3 signaling in PAH samples, and high concentration of IL-6 in the supernatants of PAH MVECs after BMP-9 stimulation, and serum from PAH patients, IL-6 was identified as an EndoMT regulator. Finally, an IL-6-neutralizing antibody completely blocked the BMP-9-mediated induction of the mesenchymal phenotype in PAH MVECs. These data suggest that BMP-9-stimulated autocrine IL-6 production in lung MVECs is involved in the pathogenesis of PAH.

Importantly, in ECs, TGF-β signaling is mediated by the TGF-β/ALK-1/Smad1,5,8 (dotted line in Fig. 1) and/or TGF-β/TβRI/Smad2,3 pathways [65]. While the former promotes the proliferation and migration of ECs, the latter is thought to antagonize and inhibit the former’s signals. Since TGF-β/ALK-1/Smad1,5,8 signaling requires the kinase activity of TβRI, the effect of TGF-β signaling on endothelial function may be regulated by the balance of both signals and, in turn, BMP-9/ALK-1 signaling could modulate TGF-β signaling in ECs, even in the pathology of PAH. The involvement of BMP-9 signaling in EndoMT has been reported [24]. Capillaries in the vasa vasorum have been reported to provide atherosclerotic sites with mesenchymal-like cells with osteogenic potential in humans [38, 66]. It has also been shown that ECs can act as additional sources of osteogenic progenitors in vascular calcification [25, 31, 40]. TNF-α and IL-1β induce EndoMT in human aortic ECs [24]. BMP-9 enhanced their response, inducing mineralization in osteoblast differentiation assay, as suggested by the upregulation of osteogenic genes, such as *osterix/OSX* and *RUNX2*. EndoMT is accompanied by the downregulation of BMPRII by TNF-α. BMPRII was also downregulated in ApoE3Leiden mice, which have previously shown the atheroprone phenotype, most likely due to human APOE and APOC1 expression, when fed a high-fat diet (HFD). Interestingly, loss of BMPRII induced by TNF-α leads to an enhanced recruitment of ACVR2A, another type II receptor for BMP-9, known as an activin receptor, in the signaling receptor complex. This is likely due to balancing downstream canonical signaling in ECs, suggested by sustained Smad1,5 activity. The JNK pathway was also downregulated by the TNF-α-induced decrease in BMP signaling in BMP-9-induced osteogenic differentiation of ECs. This was recapitulated in human advanced atherosclerotic samples, where enhanced EndoMT and the downregulation of BMPRII were observed together with sustained Smad1,5 activity.

ETS TF family members play important roles in vascular development and angiogenesis [67]. The expression of the ETS-related gene (ERG) is known to be the most abundant ETS factor in adult ECs and is essential for EC identity and protection from vascular inflammation [68, 69], as well as embryonic development and vascular stability [70, 71], and angiogenesis. ERG exerts its anti-inflammatory function in ECs by suppressing pro-inflammatory gene expression [67, 72]. ERG KD in HUVECs induced EndoMT by downregulating *CDH5*, *TIE2*, *VWF*, and upregulating *SMA*/*ACTA2* and *COL1A1* [22]. Notably, BMP signaling, which often counteracts TGF-β signaling, was suppressed, as indicated by the reduced expression of *ALK1*, *ENG* (type III receptor for TGF-β signaling), *SMAD1*, and *ID1*. ERG KD also induces the expression of TGF-β2 at the RNA and protein levels, consistent with other reports on EndoMT [44, 46, 49]. The upregulation of TGF-β2 was also observed in ECs in the liver tissue from EC-specific constitutive *Erg* hemi-deficient mice. Mechanistically, ERG and SMAD3 (direct downstream of TGF-β/TβRI signal) bind to the *TGFB2* promoter, as revealed by chromatin immunoprecipitation (ChIP) assay and ENCODE ChIP-seq data. *Erg*-deficient mice showed liver fibrogenesis. EC-specific inducible deletion of *Erg* by *Pdgfb*-CreER demonstrated increased collagen deposition, elevated SMA expression, decreased vWF expression, and increased area of PECAM1+SMA+ in the liver, suggesting that ECs underwent EndoMT in the liver of the deleted mice. These changes were reversed by the administration of a TGF-β receptor inhibitor, concomitant with the suppression of upregulated TGF-β2 expression. In liver fibrosis induced by chronic carbon tetrachloride treatment, the loss of *Erg* expression was observed in all ECs, and EndoMT was suggested by the expression of its specific markers. As *ERG* expression is downregulated by inflammatory stimuli, such as TNF-α [68] and LPS [69], treatment with etanercept, a TNF-α antagonist, rescued *Erg* expression in ECs and reversed EndoMT, leading to the suppression of fibrosis. Finally, EndoMT, indicated by PECAM1+SMA+ double-positive cells, was identified in liver fibrosis associated with alcoholic liver disease and primary biliary cirrhosis, but not in non-alcoholic steatohepatitis patients. However, the analysis of PECAM1+SMA+ cells with ERG expression in all patient samples revealed a significant negative correlation between ERG expression and EndoMT. Taken together, ERG is required to protect ECs from undergoing EndoMT in the liver in both physiological and pathological manners. Furthermore, the loss of ERG expression could be a sensitive marker of inflammation-driven fibrogenesis and a novel biomarker for EndoMT in human liver fibrosis.

ERG and FLI1 [34], which have been shown to be important for vascular development and EC function, act in an inhibitory manner on EndoMT. The combined KD of the two ETS family TFs induces EndoMT, coupled with dynamic epigenetic changes in ECs. Genome-wide analyses using ChIP-seq and microarray data revealed that ERG and FLI1 are critical transcriptional activators for EC-specific genes and repress mesenchymal-like genes through epigenetic regulation to prevent EndoMT. *miR-126*, which is known to be specifically expressed in ECs, is the key downstream target of ERG/FLI1 to regulate EndoMT. The expression of ERG and FLI1 was decreased in ECs within tumors, suggesting that EndoMT is induced in the tumor microenvironment (see the “Roles of EndoMT in Tumor EC” section). ERG and FLI1 bound to the promoter regions of *CNN1* and *TGFB2*, and the combined KD of ERG/FLI1 increased their expression, suggesting that FLI1 and ERG can modify the TGF-β/Smad pathway to protect ECs from undergoing EndoMT. Furthermore, various inflammatory cytokines, such as TNF-α, interferon-γ (IFN-γ), and IL-1β, downregulated the expression of ERG and/or FLI1 in HUVECs.

### Roles of EndoMT in atherosclerosis/arteriosclerosis

In an inflamed atherosclerosis model using casein injection in *ApoE* KO mice, HFD induced hyperlipidemia-mediated cardiac fibrosis. The reduced expression of PECAM1 and increased expression of SMA and type I collagen have been observed [26]. Chen et al. found that the KD of FRS2, an adaptor protein linking fibroblast growth factor (FGF) receptor to the MAPK, in human umbilical artery ECs (HUAECs) induced EndoMT, indicated by the upregulation of *SMA*, *TAGLN*, *CNN1*/SM-calponin, *NOTCH3*, *FSP1*, *VIM*, *FN1*, and *TGFB1* [46]. This suggests that basal FGF signaling suppresses TGF-β-mediated expression of EndoMT markers. FGF signals negatively regulate TβRI expression, which was found to be suppressed by *let-7* miRNA, suggesting that basal FGF signals maintain *let-7* expression to suppress TGF-β signaling in ECs. Furthermore, inflammatory cytokines, such as IFN-γ, TNF-α, and IL-1β, downregulate FGF receptor 1, thereby rendering ECs less responsive to FGF signals. The ratios of EndoMT cells with NOTCH3 expression, which was defined by the EC-lineage tracing method using *Cdh5*-Cre mice, were increased in the neointima and lumen of the mouse transplant arteriopathy model femoral artery injury model, and vein graft model.

Evrard et al. successfully developed endothelial-specific functional Cre recombinase using the *Scl* promoter to analyze the role of EndoMT in atherosclerosis progression using cell lineage tracing [25]. *ApoE* KO mice were fed an HFD to induce atherosclerosis. Nineteen percentages of endothelial-derived cells became positive for fibroblast associated protein (FAP) among all FAP+ cells (fibroblast-like cells) in intimal plaques of the KO mice. Among all FAP+ cells in intimal plaques, atherosclerotic mice acquired significantly higher ratios of endothelial-derived FAP+ cells. It was also shown that 7–16% of FAP+ fibroblast-like cells in the adventitia were derived from endothelial lineage cells, while fibroblasts were very rare in the media. Oxidative stress and hypoxia, both of which have been shown to promote EMT, were enhanced in atherosclerotic plaques in both the intima and adventitia. Elevated apoptosis was also observed in the atherosclerotic lesions. As a molecular mechanism, HUVECs treated with H_2_O_2_ underwent enhanced TGF-β-driven EndoMT, accompanied by acquired mesenchymal phenotypes, such as enhanced migration and invasion. HCAECs cultured under hypoxic conditions underwent EndoMT, characterized by the downregulation of *NOS3* and the upregulation of *FAP*, *TAGLN*, *CNN1*, *VCAN*, and *SNAI1/2*. The downregulation of PECAM1 was not observed by hypoxia alone, but in combination with TGF-β treatment and hypoxia. In human atherosclerotic plaques, a greater proportion of cells actively undergo EndoMT in complicated and unstable atherosclerotic plaques (classified as more advanced pathological grade). An increased proportion of FAP+ vWF+ cells was present in the ruptured plaques. When EC-derived fibroblast-like cells were compared with human fibroblasts using microarray data, the molecular signature to discriminate these cell types with regard to plaque destabilization increased MMP activity and reduced collagen expression in the former cell types, given that collagen is a hallmark of atherosclerotic plaque stability.

Helmke and colleagues found that the PECAM1+ area colocalized with the expression of fibronectin, in conjunction with collagen I deposition in the immediate vicinity of ECs in atherosclerotic lesions of *ApoE* KO mice fed an HFD [27]. The expression of *Tgfb1* as a hallmark of EndoMT and *Snai1* expression was upregulated in the aortic arches of *ApoE* KO mice fed an HFD. PECAM1+ cells co-expressed with SCA1, a mesenchymal marker/stem cell marker, were increased in atherosclerotic plaques, concomitant with elevated SMA expression. Atherosclerotic plaques rich in EndoMT cells were positive for *Hif1a* mRNA expression, which suggested increased hypoxia. Mouse cardiac ECs cultured under hypoxic conditions showed the upregulated expression of *Sca1*, *Snai1*, *Sma/Acta2*, *Tgfb1*, *Col1*, *Fn1*, and *Ctgf*. HUVECs cultured under hypoxic conditions also showed a molecular signature similar to that of mouse cardiac ECs. Similarly, there was a trend of upregulated expression of *SNAI1* and *COL1* mRNA in human atherosclerotic plaques. CD11b+ myeloid macrophages co-expressing CD11c or MHC class II, which are recruited to atherosclerotic regions, were also richer in atherosclerotic plaques. When these macrophages were co-cultured with ECs, endothelial *Snai1* expression was increased, suggesting that macrophages could induce EndoMT. During EndoMT, the mRNA expression of granulocyte-macrophage colony-stimulating factor (GM-CSF), granulocyte CSF (G-CSF), and IL10, all of which are macrophage differentiation modulators, was upregulated. Oxidized low-density lipoprotein (oxLDL) phagocytosis as a macrophage function and CD36 expression were enhanced by exposure to EndoMT. MerTK (C-Mer Proto-Oncogene Tyrosine Kinase)-mediated apoptotic cell uptake by macrophages was also promoted. These data suggest that macrophages promote EndoMT, and in turn, EndoMT cells modulate macrophage phenotype and function during atherosclerosis progression.

Cao et al. found a significant correlation between non-coding RNA (ncRNA) *H19* and TET1 in patients with coronary artery disease [32]. The expression of FN1 and VIM, target genes of TGF-β signaling in ECs, was increased in coronary artery disease patients. Since miRNA *let-7* is a regulator of TGF-β signaling during EndoMT [46], Cao and colleagues focused on the function of ncRNA *H19* in the regulation of TGF-β signaling. Although *H19* is prominently expressed in the ECs and SMCs of the prenatal rabbit aorta, its expression is negligible in adults [73]. *H19* was re-expressed in rat intima after blood vessel injury, in human left ventricle interstitial vascular structures of heart failure, and in human atherosclerotic plaques. While *H19* expression decreases in the ECs of aging mice [30], increased *H19* expression in SMCs induces aortic aneurysms in animal models. miRNA *let-7*, for which *H19* acts as a molecular sponge, inhibits the expression of TβRI. Decreased endothelial FGFR1 signaling in response to inflammatory stimuli reduced *let-7* levels and increased TβRI expression, leading to enhanced TGF-β signaling, accelerating EndoMT [46]. TET1, a member of the DNA demethylase family, is a novel target of *let-7*, and *H19* regulates TET1 expression post-transcriptionally by decreasing the bioavailability of *let-7*. TET1 regulated the expression of TβRII and TSP1 at the epigenetic level, presumably by the demethylation of the promoter of *TGFBR2* and *TSP1*. The streptozotocin (STZ) model was used to induce hyperglycemia, thereby causing endothelial dysfunction and an enhanced inflammatory response, as suggested by TNF-α upregulation. TGF-β signaling was found to be upregulated in STZ mice. However, in *H19* KO mice, TET1 expression decreased concomitantly with decreased expression of TGF-β signaling molecules, such as TβRII and TSP1, both of which involve the activation of TGF-β signaling. Decreased Smad2 phosphorylation and the downregulation of SLUG, VIM, and FN1 further suggested impaired TGF-β signaling. These results indicate that the *H19*/TET1 axis may play an important role in EndoMT in the pathogenesis of diabetes-affected arteries such as atherosclerotic inflammatory arteries.

In relation to atherosclerosis, shear stress is also an EndoMT modulator. Andueza et al. have shown the roles of disturbed flow in the induction of atherosclerosis and its association with EndoMT [29]. They performed single-cell RNA sequencing analysis and single-cell assay for transposase-accessible chromatin sequencing (scATAC-seq) using endothelial-enriched single cells from the left and right carotid arteries exposed to disturbed flow (oscillatory shear stress in the left carotid artery) and stable flow (laminar shear stress in the right carotid artery) at both acute (2-day) and chronic (2-week) phases using a mouse partial carotid ligation model. As a result, eight EC clusters were identified. The analysis of EndoMT marker genes, pathways, and pseudotime has indicated that ECs have a highly heterogeneous and plastic potential. Oscillatory shear stress (OS) induces a marked transition of ECs from the atheroprotective phenotypes to pro-inflammatory cells, mesenchymal (EndoMT) cells, hematopoietic stem cells, endothelial stem/progenitor cells, and endothelium-derived immune cell-like (EndICLT) phenotypes, as shown by Gene Ontology (GO) results related to vascular development, inflammation, apoptosis, angiogenesis, EndoMT, TGF-β pathway, and endothelial permeability. Mechanistically, in the analyses of human aortic ECs (HAECs) exposed to chronic laminar shear stress (LS) mimicking stable flow or OS mimicking disturbed flow, the expression of stable flow markers, such as KLF2/4, was found to be higher in LS, while EndICLT markers, such as *C1QC* and *C5AR1*, and EndoMT markers, such as *SNAI1* and *TAGLN*, were higher in OS. Among the eight EC clusters, three clusters (E6-E8) showed an overlapped enrichment of gene signatures in EndoMT markers at both the expression level and chromatin accessibility level (promoter accessibility). Clusters E6-E8 also showed an overlapped enrichment of gene signatures in EndICLT markers, such as *C1qa*, *C1qb*, *C5ar1*, and *Tnf*. Chronic disturbed flow induced alterations in gene expression and the chromatin accessibility in genes representing leukocyte traffic and inflammation (*Il1b*, *Il6*, *Ccl2*, *Ccl3*) and ECM regulation (*Adamts4*, *Lamb1*, *Timp3*, *Timp4*), vasoregulation, and lipid metabolism in the EC clusters, indicated by the largest difference in gene signatures between EC cluster E2 (LS type) and E8 (OS type). Transcription factor (TF) binding motifs for KLF4 were enriched in EC clusters exposed to stable flow, while TF motifs for TEF, ETV3, RELA, FOS::JUN, TEAD1, and STAT1 were enriched in EC clusters exposed to disturbed flow. Moreover, the analysis of the flow-dependent regulation of genomic regions revealed that disturbed flow markedly reduced the accessibility of the promoter region of *KLF4* and increased that of *TGFBI* (TGF-β Induced), a direct TGF-β target gene. These findings suggest that disturbed flow reprograms ECs from atheroprotective to proatherogenic phenotypes, including phenotypes of EndoMT and EndICLT.

TβRI has recently been identified as a mechanosensor for athero-prone shear stress [28]. The fluid shear stress-induced phosphorylation of Smad2 was abrogated by TβRI KD in mouse lung ECs and bovine aortic ECs. TβRI KD also induced the downregulation of *Snai1*, *Notch3*, *Fn1*, and *Cdh2*. This response was mediated by shear stress-induced binding to TβRI of Shc, a prototypical adaptor protein that is required for the transduction of receptor tyrosine kinases to downstream signaling components. The EC-specific deletion of *Shc* in mice fed an HFD immediately after partial carotid ligation significantly reduced atherosclerotic plaques, with a concomitant decrease in the ratio of SMA+, NOTCH3+, or FN1+ECs. Importantly, the response mediated by the TβRI-Shc axis was entirely independent of previously identified mechanosensors, namely, PECAM1 [74, 75] and PLXND1 [76], both of which do not activate Smad2.

### Roles of transient EndoMT in EC plasticity

Tombor et al. elucidated EC plasticity by analyzing transient gene reprogramming in ECs and EC-derived cells after MI induced by left anterior descending coronary artery ligation [8]. scRNA-seq analyses of non-cardiomyocyte fractions were performed at days 0, 1, 3, 5, 7, 14, and 28 post-MI. Among the 19 clusters, four EC clusters were identified as well as 5 clusters of monocytes, six clusters of fibroblasts, and single clusters of SMCs, T cells, and B cells. The ratio of ECs was potently reduced on days 1 to 7, and recovered after day 14. The ratio of macrophages potently increased on day 1, was sustained at day 7, and was reversed to a homeostatic level thereafter. The ratio of mesenchymal cells remained relatively stable, but decreased between days 1 and 7. The relative numbers of ECs, which have an inflammatory gene signature defined by *Il1b* (majority), *Tnfa*, and *Il6* (minority), peaked at days 1 to 3, and then gradually continued to decrease until day 28. Between days 1 and 7, the mesenchymal gene signature was upregulated, as suggested by the increased expression of *Fn1*, *Mmp14*, *S100a4*, *Vim*, *Tnc*, *Col1a1*, *Col3a1*, *Serpine1*, and *Snai1*. The altered gene expression coincided with the upregulated gene signature of fatty acid metabolism and cell cycle/proliferation. These changes were only sustained on days 1 to 7, returning to the baseline levels after day 14, suggesting that ECs acquired a transient mesenchymal activation (denoted as EndMA by the authors, but also known as “partial EndoMT”) (Fig. 3). EndMA+ cells that co-express EC genes and mesenchymal genes were compared to EndMA– cells that lack mesenchymal gene expression. EndMA+ ECs showed high levels of expression for both mesenchymal and ECM genes, an enriched expression of genes associated with epithelial–mesenchymal expression (EMT gene expression), and GO terms associated with ECM organization, PDGF binding, collagen synthesis, and organization. On the other hand, EndMA– ECs showed high levels of expression for genes involved in fatty acid signaling, EC-enriched TFs, and GO terms associated with VEGF and VEGF-receptor response, vascular development, and negative regulation of EC proliferation. Focusing on metabolic gene signatures, EndMA+ cells showed a high expression of glycolysis genes but reduced expression of fatty acid signaling and TCA cycle genes, suggesting a metabolic switch from fatty acids toward glycolytic metabolism during EndMA. Importantly, EC lineage-tracing analyses using the *Cdh5*-CreERT2 system further confirmed the transient activation of mesenchymal genes. This was further recapitulated in in vitro studies, where the upregulated expression of *TAGLN* and *CNN1/SMCalponin* was reversed upon the removal of TGF-β (Fig. 3).

### Roles of EndoMT in tumor EC

The tumor microenvironment is composed of various components, including cancer cells, tumor vessels, CAFs, and inflammatory cells. Various cancer cell types express high levels of TGF-β, which induces the EndoMT. TGF-β-induced EndoMT is enhanced by inflammatory cytokines, such as TNF-α and IL-1β, leading to the formation of CAFs. Multiple types of human primary ECs underwent EndoMT in response to TGF-β and TNF-α, which was accompanied by the increased and decreased expression of mesenchymal cell and EC markers, respectively [49]. EndoMT cells co-treated with TGF-β and TNF-α highly expressed both TGF-β2 and activin A, suggesting that TNF-α enhanced TGF-β-induced EndoMT by enhancing the TGF-β family signals. Furthermore, oral carcinoma cells underwent EMT in response to humoral factors produced by the EndoMT cells, suggesting that the cells function as tumor-promoting CAFs. This EndoMT-driven EMT was blocked by a TGF-β-neutralizing antibody, suggesting that TNF-α enhances TGF-β-dependent EndoMT, which contributes to tumor progression.

EndoMT has been shown to be an important driver of neointima formation in a murine transplant arteriopathy model [46], in which endogenous FGF signals play a critical role in inhibiting EndoMT. In tumors, Xiao et al. found that TGF-β treatment induces FGF2 expression and secretion, which in turn inhibits TGF-β signaling in a positive feedback loop in TECs isolated from spontaneous mammary tumors in mice [35]. Conditioned media harvested from TGF-β-treated TECs contained high levels of FGF2, which play a role in suppressing mesenchymal gene expression. However, various TECs even in the same model of tumors displayed a wide range of EC phenotypes, suggesting EC heterogeneity, including TECs with high and low levels of SMA expression (myofibroblastic and non-myofibroblastic TECs, respectively). Akatsu et al. also examined the effect of endogenous FGF signals in TECs isolated from human metastatic melanoma xenografted tumors [48]. Autocrine or paracrine FGF signals in TECs inhibit the TGF-β-induced endothelial–myofibroblast transition (EndoMyoT), leading to the suppressed formation of contractile myofibroblast cells. However, the FGF signals can also collaborate with TGF-β in accelerating the formation of active fibroblastic cells with migratory and proliferative properties, suggesting the action of giving rise to heterogeneous mesenchymal TECs. In a xenograft model, TECs treated with TGF-β were more competent in promoting tumor growth than TECs treated with TGF-β and FGF2. Mechanistically, Elk1 mediated the FGF2-induced inhibition of EndoMyoT through the inhibition of the TGF-β-induced transcriptional activation of the SMA promoter by MRTF-A (shown as light green in Fig. 2). The results of the two groups suggest that TGF-β and FGF2 oppose and cooperate with each other during the formation of myofibroblastic and non-myofibroblastic cells from TECs, determining the characteristics of EC-derived mesenchymal cells in the tumor microenvironment.

Adrenomedullin (AM) signaling plays an important role in cardiovascular homeostasis [77]. AM is required for angiogenesis and has anti-inflammatory, antioxidative, and anti-fibrotic properties. AM acts through a G protein-coupled receptor, calcitonin receptor-like receptor (CLR). The specificity of CLR for its ligands is dependent on three kinds of receptor activity-modifying proteins, RAMP1, 2, and 3. Using the EC-specific inducible deletion of *Ramp2* KO mice (R2cKO, hereafter), the role of the vascular AM-RAMP2 system in both tumor angiogenesis and metastasis was examined. The R2cKO mice showed poor angiogenesis in tumors formed by transplantation with sarcoma or melanoma cells, as suggested by decreased tumor weight and decreased expression of endothelial markers, such as *PECAM1*, *VEGFR2/KDR*, and *VEGF* [36]. Tumor metastasis was found to be enhanced in the R2cKO mice. The permeability of tumor vessels was enhanced, as well as the abnormal growth of SMA+ mesenchymal cells within vascular walls, in the R2cKO mice, whereas the number of PECAM1+ECs was decreased, which strongly suggests an EndoMT-like change in TECs. Mechanistically, when HUVECs were co-cultured with cancer cells isolated from primary tumors formed in the R2cKO mice, enhanced stress fiber formation, which is characteristic of EndoMT cells, was observed. TGF-β-treated HUVECs showed a similar phenotype, which was reversed by pre-treatment with AM. When isolated pulmonary ECs from the R2cKO mice were treated with TGF-β, the EndoMT-like phenotype was enhanced, as indicated by the upregulated expression of FSP1. The prominent invasion of the lungs by macrophages was observed in R2cKO mice. In addition, enhanced oxidative stress and the upregulation of inflammatory cytokines, such as *Il6*, *Tnfa*, and *Tgfb1*, were detected in the lungs of R2cKO mice. The expression of *S100a8/9*, which has been identified as a tumor metastasis-enhancing factor in distant organs [78, 79], and that of the downstream target serum amyloid A3 (SAA3) was strongly upregulated in the R2cKO lung. Finally, the EC-specific overexpression of *Ramp2* in mice suppressed spontaneous lung metastasis after transplantation of B16 melanoma cells. Moreover, transgenic mice showed greater survival in the lung metastasis model. These results suggest that vascular integrity regulated by the AM-RAMP2 system could be a good therapeutic target for suppressing tumor metastasis.

The cyclooxygenase-dependent production of prostaglandins is crucial for tumor angiogenesis and growth [80, 81]. Omori and colleagues found that COX2/PTGS2 was significantly upregulated in melanoma ECs compared to normal lung EC [37]. The concomitant upregulation of *Il1b*, *Il4*, hematopoietic prostaglandin D synthase (*Hpgds*), prostaglandin I synthase (*Ptgis*), Mmp9, and *Vcam1* was also observed in melanoma ECs. In particular, lipocalin-type prostaglandin D synthase (*Ptgds*/L-PGDS) expression was markedly increased in melanoma ECs and ECs in Lewis lung carcinoma cell-driven tumors. The upregulation of L-PGDS was suppressed by the inhibition of IL-1 and TNF-α. EC-specific, as well as the systemic deletion, of *Ptgds* in mice increased the tumor size of melanomas, which was most likely due to decreased apoptotic tumor cells induced by decreased L-PGDS expression. The EC-specific deletion of *Ptgds* in mice has been found to increase vascular permeability, angiogenesis, and vascular perfusion. Moreover, the deletion also led to the enhancement of EndoMT, indicated by an increased ratio of PECAM1+SMA+ ECs. Mechanistically, the expression of *SMA/ACTA2* and *TGFB1* was increased in melanoma culture-treated HUVECs when L-PGDS was inhibited. These data suggest that tumor cell-derived inflammatory cytokines increase L-PGDS expression and subsequent PGD_2_ production in TECs, leading to a negative regulation of tumorigenic changes in TECs.

Choi et al. previously reported on radiation-induced EndoMT. Trp53 is a key regulator of radiation responses in ECs. They found that Trp53 is a positive regulator and TβRII is a negative regulator of radiation-induced EndoMT in in vitro studies using siRNA-treated HUVECs [15]. Irradiation usually temporarily suppresses the growth of Kras- and Trp53-driven spontaneous lung tumors (KP tumors), but delayed tumor regrowth was observed. During radiation resistance, the *Tie2*-Cre-mediated EC-specific deletion of *Trp53* significantly delayed tumor regrowth. Conditional KO (Trp53cKO, hereafter) mice showed a decrease in the progression of EndoMT and elevated collagen deposition, as suggested by a reduced number of PECAM1+SMA+ cells. In addition, the ratios of SMA+NG2+ pericytes in tumor vessels decreased in Trp53cKO mice, suggesting much leakier and less functional tumor vessels. On the other hand, enhanced EndoMT and collagen deposition were observed, and tumor regrowth was accelerated in the irradiated KP tumors of EC-specific *Tgfbr2* KD (Tgfbr2 conditional KD, Tgfbr2cKD) mice, suggesting that tumors acquire radioresistance. Moreover, Trp53cKO overcame Tgfbr2cKD-induced radioresistance and Tgfbr2cKD-enhanced tumor regrowth. Cancer cells that acquire radioresistance exhibit cancer stem cell-like characteristics. In radioresistant cancer stem cells (CSCs) during tumor regrowth, areas including cancer cells positive for CSC markers, such as aldehyde dehydrogenase, CD44, and CD133, did not significantly increase in irradiated tumors compared to non-irradiated ones. However, the CD44 variant CD44v6+ cell population showed the strongest increase after radiotherapy. Among CD44 variant isoforms, CD44v6 is a colorectal CSC marker required for the metastatic potential of CSCs [82]. CD44v6 expression is largely restricted to the advanced stages of pancreatic cancer and more prevalent in metastatic than in non-metastatic adenocarcinomas [83]. CD44v6+ cells were more abundant in irradiated metastatic tumors than in non-irradiated metastatic tumors. Lung metastatic nodule numbers from KP tumors were significantly decreased in Trp53cKO mice. Irradiation downregulated EC-specific genes and upregulated fibroblast-specific genes in KP tumor ECs isolated from WT, but not Trp53cKO mice. Osteopontin (OPN), which is known to interact with CD44, was identified by human soluble receptor array analysis of conditioned medium from human pulmonary microvascular ECs after irradiation, and RNA-seq analysis of upregulated genes in Tgfbr2cKD mice or TβRII-KD HUVECs. OPN correlates with proliferating CD44v6+ cancer cells in hypoxic areas. In Tgfbr2cKD mice, anti-OPN antibodies reduced tumor growth after irradiation. Activated macrophages are often classified as pro-inflammatory M1 macrophages or anti-inflammatory M2 macrophages. In cancer context, polarization toward the M2 phenotype showed strong correlation with the aggressiveness of some cancers, and even with anti-tumor treatment resistance [84, 85]. EndoMT modulates the M1/M2 polarization of the tumor-associated macrophage (TAM) populations after radiotherapy. Trp53cKO attenuated the M2 polarization of SDF1+ TAMs, while TECs through EndoMT showed strongly increased expression of CXCR4, a specific receptor for SDF1. Tumors in WT mice decreased and increased M1-type and M2-type macrophages, respectively, while those in Trp53cKO mice showed reversed polarization of M1/M2 subtypes. Mechanistically, the co-culture of WT ECs with F4/80+ macrophages induced a significant increase in the CD206+ M2 subtype. In terms of its clinical relevance, tumor SMA+PECAM1+ vessels and coverage with SMA+NG2+ pericytes were correlated in tumor vessels in patients who underwent radiotherapy. The higher percentage of residual tumor cells after irradiation was CD44v6+ in patients than in non-irradiated patients. Higher percentages of CD44v6+ CSCs in irradiated cancer tissues were positive for OPN compared to non-irradiated controls. In summary, in addition to giving rise to the formation of CAFs, EndoMT not only functionally weakens the endothelial barrier of the tumor vasculature and facilitates metastatic dissemination but also modulates the tumor microenvironment by affecting the immune cell response and stemness of cancer cells. Thus, EndoMT is considered a potential novel target for anticancer therapies [86].

Choi et al. also found that HSPB1 expression is abundant in TECs in lung tumors, which is spontaneously induced by oncogenic *Kras* and *Trp53* deficiency [14, 87]. The intranasal administration of short-hairpin HSPB1 (shHSPB1) significantly suppressed the number of tumorigenic lung nodules concomitant with enhanced collagen deposition, increased FSP1+ regions, and TGF-β1 expression. Furthermore, samples from various types of human lung cancer, including small-cell, large-cell, and squamous cell carcinoma and adenocarcinoma, showed high levels of HSPB1 expression in TECs. Notably, a significantly higher expression in non-small cell lung cancer (NSCLC) was observed in non-fibrotic regions than in fibrotic regions, as well as higher ratios of EndoMT in HSPB1-negative vessels compared to HSPB1+ vessels. These data suggest that the low expression of HSPB1 correlates with enhanced EndoMT and fibrosis in human lung cancers, such as NSCLC.

### Roles of EndoMT in autophagy/mitophagy and ER stress

Impaired autophagy correlates with EndoMT in exacerbating organ fibrosis. When ATG5, a well-known crucial autophagy-related protein, was knocked down by siRNA in human microvascular ECs (HMVECs), EndoMT was induced, suggested by the downregulation of EC markers, the upregulation of mesenchymal cell markers, and the elevated phosphorylation of SMAD3 [9]. EndoMT was not inhibited by a TGF-β-neutralizing antibody, suggesting that EndoMT was not mediated largely by TGF-β. Instead, IL-6 was significantly upregulated in ATG5-knocked HMVECs. EndoMT was almost completely blocked by an IL-6-neutralizing antibody. Monitoring by the cell lineage tracing system, the *Cdh5*-Cre-mediated deletion of ATG5 in mice induced renal fibrosis, which was further enhanced by HFD. Endothelial autophagy defects are characteristic of patients with type 2 diabetes. The mice also showed metabolic disorders, such as insulin resistance, increased HbA1c levels, increased systolic blood pressure, and decreased diastolic blood pressure, all of which were reversed by treatment with an IL-6-neutralizing antibody, which also ameliorated renal fibrosis and glomerular hypertrophy.

Chronic renal graft dysfunction (CAD), formerly known as chronic allograft nephropathy, is a multifactorial condition associated with progressive renal interstitial fibrosis. CAD is morphologically characterized by inflammation, progressive interstitial fibrosis, tubular atrophy, and glomerular sclerosis. Gui et al. found that the expression of EndoMT markers was notably higher in CAD patients than in non-CAD patients [23]. Based on RNA-seq data from both CAD patient groups and non-CAD groups, they also found that 14 autophagy-related genes as candidate markers showed a stronger correlation in CAD disease progression stages. Among them, the expression of ATG16L, which is involved in the early step of autophagy, decreased with the progression of CAD, characterized by the progression of EndoMT and fibrosis. In the CAD patient groups, low expression of MAP1LC3 (LC3), which is critical for autophagosome formation and the activation of autophagic flux, was correlated with poor survival. This was further confirmed by the downregulation of LC3 in the serum. Mechanistically, ATG16L KD inhibited autophagy in human renal glomerular ECs (HRGECs), characterized by a decrease in LC3B-II formation. The KD of ATG16L expression also induced EndoMT expression. A large amount of collagen fibers accumulated in the glomerular and renal interstitium in rat renal transplant models of chronic rejection, coinciding with the high expression of FN1, and SMA and the low expression of PECAM1. RNA-seq in HRGECs with ATG16L KD revealed that the NF-κB pathway was one of the significantly activated pathways. NF-κB was stabilized, and its nuclear translocation was sustained by decreased degradation in ATG16L KD. A significant increase in p65/RELA was observed in the renal allografts of patients with CAD. In vitro analysis using HRGEC showed that EndoMT was induced by IL-1β or IL-6, and most strikingly by TNF-α, and that EndoMT was blocked by their specific inhibitors. Taken together, autophagy plays a cytoprotective role during renal transplantation by inhibiting EndoMT.

Kaposi’s sarcoma-associated herpesvirus (KSHV) is the causative agent of Kaposi’s sarcoma, an aggressive neoplasm that often occurs in immunocompromised patients. Santarelli et al. found that KSHV induced KS pathogenesis through EndoMT in ECs [45]. KSHV-infected HUVECs showed spindle cell morphology as well as the downregulation of EC markers and the upregulation of mesenchymal cell markers, suggesting that KSHV causes EndoMT. Mechanistically, the phosphorylation of mTOR and 4EBP1 was elevated, suggesting the activation of the mTOR pathway. mTOR is also known to be a master negative regulator of autophagy/macroautophagy. The lapidated form of LC3II was detected at a lower level, suggesting that KSHV infection reduced basal macroautophagy in HUVECs. The serine phosphorylation of ULK1 and degradation of the mitochondrial matrix protein hydroxyacyl-CoA dehydrogenase, both of which are negative indicators of autophagy, were observed in infected HUVECs. These results suggest that both bulk macroautophagy and mitophagy are impaired by KSHV infection. KSHV also increased intracellular ROS and activated the ER stress/unfolded protein response (UPR) in HUVECs. The KD of RAB7, the last-step regulator of macroautophagy, inhibited macroautophagy but induced SNAI1 expression. KSHV-induced SNAI1 expression was inhibited by metformin, a protein kinase AMP-activated catalytic subunit alpha 2 activator, which is able to induce macroautophagy. It has been shown that the KSHV-mediated activation of ATF4 promotes the release of the pro-inflammatory chemokine CCL2. Increased production of CCL2 and upregulated expression of SNAI1 in the infected HUVECs, which was blocked by an inhibitor of PERK, a component of the PERK/eIF2α /ATF4 axis downstream of the UPR, suggesting that UPR activation promoted KSHV-driven sarcomagenesis by increasing the release of CCL2 and promoting EndoMT.

### Roles of EndoMT in lymphatic/lymphatic-like vessels

Multiple characteristics in inflammation are shared between aging and age-related diseases. Aging is accompanied by a chronic, sterile, low-level inflammation called inflammaging [88]. A decrease in the lymphatic vessel network has been reported in human skins aged by UV irradiation-induced inflammation [89], and a correlation between decreased lymphatic vessel density and the functional decline has been reported in aged mice [90]. Thus, in previous studies, decreased functions of lymphatic vessels have been strongly correlated with changes in EC identity mainly associated with aging, and lymphatic EndoMT has often been analyzed from the perspective of aging and its associated functional decline.

The lumen of lymphatic vessels is lined with lymphatic ECs (LECs). LECs are defined as the expression of LEC markers such as LYVE1, Prox1, PDPN, and FLT4 (also known as VEGFR3). LYVE1 was first identified as a LEC marker and is now widely used. Prox1 is a master regulator of lymphatic development and is required for its cellular identity even in adults. Both *PDPN* and *FLT4* are downstream target genes of Prox1.

KSHV can also infect differentiated primary LECs. Spindle cells with expression of heterogeneous mesenchymal markers have been reported in Kaposi sarcoma lesions [91–93]. A three-dimensional (3D) cell culture model was established to provide a more physiological environment for the cells [42] and is useful for analyses on endothelial capillary sprouting. KSHV induced LECs to undergo EndoMT in 3D culture, suggested by induction of significantly higher expression of mesenchymal genes such as *TAGLN*, *CNN1*, *PDGFRB*, *S100A4*, *VIM*, *COL1A1*, *CDH2*, compared to LECs in 2D culture. The infected LEC also showed significant downregulation of *PROX1*, and *FLT4*. Induction of EndoMT by KSHV was found to be suppressed by inhibition of Notch signaling pathway using DAPT, a γ-secretase inhibitor or Fc chimeric receptor fused to Dll4, which is a ligand for Notch. VEGF-A has been shown antagonized by Notch signaling, and VEGF-C is known to bind VEGFR3 to exert a lymphangiogenic function. Treatment of VEGF-A and VEGF-C showed extensive sprouting of KSHV-infected LEC, but VEGF-A/C-treated spheroids did not induce mesenchymal gene expression such as SMA and FN1, suggesting that EndoMT is blocked by VEGF-A/C in 3D-cultured KSHV-infected LECs. KSHV-specific viral proteins, vGPCR and vFLIP, have previously been shown to upregulate DLL4 and JAG1, respectively, both of which are positive regulators of Notch signaling. Forced expression of both proteins induced mesenchymal genes as well as Notch target genes, *HEY1* and *HES1*. Transcriptome analysis of KSHV-infected LEC revealed upregulation of tissue inhibitor of metalloproteinases, *TIMP1* and *TIMP2*. Among them, TIMP2 potently inhibited sprouting, suggesting the involvement of MT-MMPs in the sprouting since TIMP2 reduces the activity of membrane-bound MT-MMPs. Knockdown of MT1-MMP/MMP14 dramatically reduced the sprouting/invasive property of KSHV-infected LEC with concomitant suppression of *PDGFRB* as well as *TAGLN*. Moreover, KD of PDGFRB inhibited the sprouting of the spheroids, and suppressed expression of *TAGLN* and *VIM*, suggesting that PDGFRB regulates mesenchymal genes in KSHV-infected LECs.

Lymphatic EndoMT is also observed in lymphedema. In the tail lymphedema model in mice by removing lymphatic vessel-included tail dermis, LYVE1+SMA+ LECs emerged in regenerating lymphatic vessels during lymphedema-associated lymphangiogenesis [33]. FGF2 depletion acted synergistically with TGF-β to induce EndoMT. Mechanistically, Erk and MEK, both of which are known to be downstream molecules activated by FGF signals, induced phosphorylation of the linker domain of Smad2, thereby inhibiting Smad nuclear accumulation and suppressing EndoMT.

Schlemm’s canal (SC) is a specialized vascular structure showing lymphatic endothelial-like phenotype due to expression of the Prox1 TF [94, 95]. Accumulated attention has been paid in recent years since the dysfunction of SC leads to glaucoma development. Park et al. reported aging correlated with dysfunction of SC, with co-expression of SMA and PECAM1 and downregulation of Prox1 expression, suggesting SC caused EndoMT with aging. Elucidating a molecular mechanism of EndoMT in SC will be strongly expected to understand the pathology of glaucoma.

Dermal LECs undergo EndoMT, suggested by downregulation of *LYVE1*, *PROX1*, *ANG2*, and upregulation of *TAGLN*, *FN1* [41]. Lymphatic EndoMT showed increased motility, similarly to KSHV-infected LEC. Moreover, when microvessels were formed in tubes of collagen gels using dermal LECs, the permeability of the TGF-β-treated vessels was dramatically enhanced. MS-1 cells, mouse-derived blood vascular ECs, have been previously shown to undergo EndoMT through both Smad and non-Smad pathways, in the downstream of TGF-β signaling [96]. Similarly, TGF-β-induced EndoMT in LEC was suppressed by siRNA for *SMAD2* as well as *MKL1* encoding MRTF-A, which is downstream of Rho A/ROCK pathway (one of non-Smad pathways). Under inflammatory conditions such as stimulation of TNF-α, dermal LECs enhanced TGF-β-induced EndoMT by enhancement of up- and downregulation of EndoMT markers except for a few markers. Mechanistically, TGF-β-induced EndoMT increased the production of endogenous activin A by HDLECs, which leads to the long-lasting activation of Smad2,3 signals. Moreover, the expression of *FST* encoding Follistatin, an inhibitor of activin A, decreased during EndoMT. These results suggest activin signals endogenously enhanced by TGF-β-treated LECs lead to TGF-β-independent phosphorylation of Smad2 and further enhanced EndoMT. Human-aged skin tissues showed elevated ratios of EndoMT, suggested by LYVE1+TAGLN+ co-expressed LECs. The EndoMT ratio defined by these two markers could be a promising method for evaluating dermal lymphatic function as well as estimating skin function and skin aging.

### Heterogenic EndoMT response in various ECs

As described above, a wide variety of inflammation-mediated EndoMTs have been reported in various animal models and various ECs derived from different organs and tissues. The phenotypes of ECs differ from organ to organ, suggesting heterogeneity in EC function. Pinto et al. clarified distinct responsiveness during SNAI1/TGF-β-mediated EndoMT in human primary ECs of different origins (HUVECs, HPAECs, HAECs, and HCAECs) [39]. Interestingly, HUVECs, which are the most frequently used ECs, showed the least responsiveness to SNAI1 overexpression in the induction of mesenchymal markers and repression of EC markers. In addition, even with TGF-β treatment, SNAI1-overexpressed HUVECs did not induce *TAGLN* expression. As for the characteristics that tend to cause EndoMT, HAEC and HCAEC were more responsive than the other two ECs. From the point of view on the requirement of EMT/EndoMT TFs for EndoMT, SNAI1 is required for TGF-β-induced mural cell differentiation from embryonic stem cell-derived ECs [4]. TGF-β induces *Snai1* expression in ECs, and the KD of Snai1 reduces the TGF-β-induced upregulation of *Sma/Acta2*, *Cnn1*, and *Tagln* and the downregulation of *Cldn5*. In contrast, *Snai1* is not required for TGF-β-induced EndoMT in MS-1 cells derived from mouse pancreatic ECs, although *Snai1* is upregulated by TGF-β [96]. This implies that other EndoMT TFs are responsible for the EndoMT or compensation by other EndoMT TFs for loss of SNAI1 expression. TIE1 deficiency induces EndoMT with the upregulation of *SNAI2* but not *SNAI1* in human microvascular ECs (HMVECs) [5]. The KD of SNAI2 reduces the TIE1 KD-induced upregulation of *COL1A1* and *FSP1/S100A4*, and the downregulation of *CDH5* and PECAM1. The VEGF-induced decrease in CLDN5 expression was blocked by SNAI2 KD in HUVECs [97]. Upregulated SNAI2 expression was observed in angiogenic tumor vessels in an orthotopic, syngeneic mouse colorectal cancer model, as well as fibrin gel angiogenesis assays in vitro [43]. SNAI2 KD disrupted the EC sprouting in these models. SMA expression is upregulated in hypoxia-induced vascular remodeling in pulmonary arterioles in mice. EC-specific deficiency of *Twist1* using *Tie2*-Cre mice suppresses remodeling with a decrease in SMA expression [6]. Taken together, functionally critical EndoMT TFs vary among EC types.

## Conclusions

Since TGF-β and inflammatory signaling factors induce or enhance EndoMT, simply blocking TGF-β signaling may not be sufficient for inhibiting EndoMT, which would result in ameliorating its associated diseases. Blocking inflammation signals or the combined blocking of TGF-β and inflammation signals may be more effective for inhibiting EndoMT, based on accumulating lines of evidence on the roles of inflammation-mediated EndoMT. Importantly, rapidly increasing attention is being focused on the involvement of inflammation and EC response to inflammation, including inflammation-mediated EndoMT in COVID-19 pathogenesis. As mentioned in this review, although various EndoMT regulators have been identified, differences in response to cytokine stimuli due to heterogeneity of organotypic ECs need to be considered. The development of therapies for EndoMT-related diseases by targeting these regulators is now strongly expected in the near future.

## Abbreviations

ActRI: Activin type I receptor
ActRII: Activin type II receptor
ALK-1: Activin receptor-like kinase-1
ALK-5: Activin receptor-like kinase-5
AM: Adrenomedullin
BMDM: Bone marrow-derived macrophage
BMP: Bone morphogenetic protein
BMPRII: BMP type II receptor
CAD: chronic renal graft dysfunction
CAF: Cancer-associated fibroblast
ChIP: Chromatin immunoprecipitation
CSC: Cancer stem cell
CSF: Colony-stimulating factor
EC: Endothelial cell
ECM: Extracellular matrix
EMT: Epithelial-mesenchymal transition
ENCODE: Encyclopedia of DNA Elements
EndICLT: Endothelium-derived immune cell-like
EndoMT: Endothelial-mesenchymal transition
EndoMyoT: Endothelial-myofibroblast transition
ERG: ETS-related gene
Erk: Extracellular signal-regulated kinase
FAP: Fibroblast associated protein
FGF: Fibroblast growth factor
FSP1: Fibroblast specific protein 1
GSEA: Gene set enrichment analysis
HAEC: Human aortic EC
HCAEC: Human coronary artery EC
HFD: High-fat diet
HMVEC: Human microvascular EC
HPAEC: Human pulmonary artery EC
H-PGDS: Hematopoietic prostaglandin D synthase
HPMEC: Human pulmonary microvascular EC
HRGEC: Human renal glomerular EC
HUAEC: Human umbilical artery EC
HUVEC: Human umbilical vein EC
ICAM1: Intracellular adhesion molecule-1
IFN-γ: Interferon-γ
IL-1β: Interleukin-1β
JNK: c-Jun NH2-terminal kinase
KD: Knockdown
KO: Knockout
KSHV: Kaposi’s sarcoma-associated herpesvirus
LacZ: β-Galactosidase
LAMB1: Laminin subunit beta 1
LC3: Microtubule-associated protein 1 light chain 3
LEC: Lymphatic EC
L-PGDS: Lipocalin-type prostaglandin D synthase
LPS: Lipopolysaccharide
LS: Laminar shear stress
MAEC: Mouse aortic EC
MAPK: Mitogen-activated protein kinase
MCT: Monocrotaline
MEK: MAPK kinase
MI: Myocardial infarction
MMP: Matrix metalloproteinase
ncRNA: Non-coding RNA
OPN: Osteopontin
OS: Oscillatory shear stress
PAH: Pulmonary arterial hypertension
PDGF: Platelet-derived growth factor
PDGFRβ: PDGF receptor β
PERK: Protein kinase R-like endoplasmic reticulum kinase
PH: Pulmonary hypertension
PI3K: Phosphoinositide 3-kinase
ROS: Reactive oxygen species
SARS-CoV-2: Severe acute respiratory syndrome coronavirus 2
SC: Schlemm’s canal
SMC: Smooth muscle cells
STZ: Streptozotocin
TAM: Tumor-associated macrophages
TβRI: TGF-β type I receptor
TβRII: TGF-β type II receptor
TF: Transcription factor
TGF-β: Transforming growth factor-β
TGFBR1: TGF-β type I receptor
TGFBR2: TGF-β type II receptor
TIMP: Tissue inhibitor of metalloproteinases
TNF-α: Tumor necrosis factor-α
UPR: Unfolded protein response
VCAM1: Vascular cell adhesion molecule-1
VEGF: Vascular endothelial growth factor
VEGFR2: VEGF receptor 2
VEGFR3: VEGF receptor 3
vFLIP: Viral FLICE inhibitory protein
vGPCR: Viral G protein-coupled receptor
